# Transcription analysis of the porcine alveolar macrophage response to porcine circovirus type 2

**DOI:** 10.1186/1471-2164-14-353

**Published:** 2013-05-27

**Authors:** Wentao Li, Shuqing Liu, Yang Wang, Feng Deng, Weidong Yan, Kun Yang, Huanchun Chen, Qigai He, Catherine Charreyre, Jean-Christophe Audoneet

**Affiliations:** 1 Division of Animal Infectious Disease, State Key Laboratory of Agricultural Microbiology College of Animal Science and Veterinary Medicine, Huazhong Agricultural University, Wuhan, Hubei 430070, China; 2 Merial SAS, Lyon 69007, France

## Abstract

**Background:**

Porcine circovirus type 2 (PCV2) is the causal agent of postweaning multisystemic wasting syndrome (PMWS), which has severely impacted the swine industry worldwide. PCV2 triggers a weak and atypical innate immune response, but the key genes and mechanisms by which the virus interferes with host innate immunity have not yet been elucidated. In this study, genes that control the response of primary porcine alveolar macrophages (PAMs), the main target of PCV2, were profiled *in vitro*.

**Results:**

PAMs were successfully infected by PCV2-WH strain, as evidenced quantitative real-time polymerase chain reaction (qPCR) and immunofluorescence assay (IFA) results. Infection-related differential gene expression was investigated using pig microarrays from the US Pig Genome Coordination Program and validated by real-time PCR and enzyme-linked immunosorbent assay (ELISA). Microarray analysis at 24 and 48 hours post-infection (HPI) revealed 266 and 175 unique genes, respectively, that were differentially expressed (false discovery rate <0.05; fold-change >2). Only six genes were differentially expressed between 24 and 48 HPI. The up-regulated genes were principally related to immune response, cytokine activity, locomotion, regulation of cell proliferation, apoptosis, cell growth arrest, and antigen procession and presentation. The down-regulated genes were mainly involved in terpenoid biosynthesis, carbohydrate metabolism, translation, proteasome degradation, signal transducer activity, and ribosomal proteins, which were representative of the reduced vital activity of PCV2-infected cells.

**Conclusions:**

PCV2 infection of PAMs causes up-regulation of genes related to inflammation, indicating that PCV2 may induce systematic inflammation. PCV2 persistently induced cytokines, mainly through the Toll-like receptor (TLR) 1 and TLR9 pathways, which may promote high levels of cytokine secretion. PCV2 may prevent apoptosis in PAMs by up-regulating SERPINB9 expression, possibly to lengthen the duration of PCV2 replication-permissive conditions. The observed gene expression profile may provide insights into the underlying immunological response and pathological changes that occur in pigs following PCV2 infection.

## Background

Porcine circovirus 2 (PCV2) is a small, non-enveloped, single-stranded and closed-circular DNA virus that has been identified as the primary cause of postweaning multisystemic wasting syndrome (PMWS) in pigs. PMWS primarily affects pigs between the ages of five and 18 weeks. The clinical signs include progressive weight loss, jaundice, wasting, and respiratory disease, accompanied by increased mortality. The mortality rates of PMWS vary from 1-2%, but may reach up to 30% in complicated cases. PMWS has become endemic in many swine-producing countries, including China, Canada, the United States, Venezuela, Australia, and the United Kingdom [1-3].

DNA microarray technology, in combination with bioinformatics, has emerged as an efficient high-throughput tool that offers great advantages in the study of the genomic expression profiles of cells and host-microbe interactions [4]. This technology provides the ability to determine gene expression levels of thousands of different genes simultaneously. Indeed, high density gene arrays have already revealed the status of host gene expression following infection by several viruses and bacteria, including influenza A virus [5], monkeypox virus [6], Epstein-Barr virus [7], *Streptococcus suis*[8], and *Haemophilus parasuis*[9].

The major target cells of PCV2 are the porcine monocyte/macrophage lineage cells (MLCs) [10-13]. One type of MLC, porcine alveolar macrophages (PAMs), are the primary responders of the pulmonary innate immune system and defend against various pathogens [14]. While PAMs are known to be involved in PCV2 defense, the underlying molecular mechanisms of PCV2 pathogenicity remain to be completely elucidated. Furthermore, an immunosuppressive role has been characterized for PCV2 in porcine circovirus diseases (PCVD) [15,16], but little is known about the genes that are involved in this process. The presence of granulomatous inflammation and multinucleated giant cells in PMWS pigs [16] suggests a significant role for secreted cytokines and other immune-related signaling factors. Previous studies have investigated the cytokine and chemokine gene expression profile of PMWS by transcriptomic and genomic analyses. For example, the cytokine mRNA expression profile in lymph nodes of PMWS pigs was reported [17], as was the global transcriptome profile of colostrums-deprived piglets experimentally infected with PCV2 [18,19]. The genomic expression profile in lymph nodes of lymphoid-depleted PCV2-infected pigs was also reported [20]. However, these results can not fully illustrate the pathogenesis of PCV2.

Therefore, the objective of the present study was to analyze the PCV2-induced gene expression profiles in isolated PAMs *in vitro* in order to reveal specific mechanisms of the host innate immune response to PCV2. The temporal gene expression profiling using a two-channel microarray carrying thousands of genes was carried out with PAMs at 24 and 48 hours post-infection (HPI) to discriminate PCV2-induced early and late changes in gene expression.

## Results

### Confirmation of PCV2 infection in PAMs

**Figure 1:**
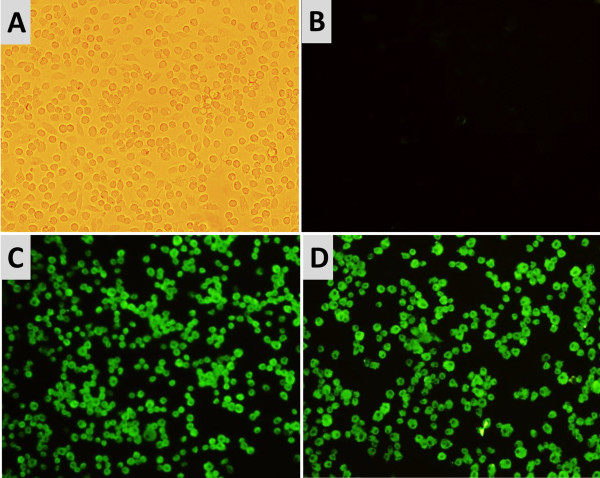
**Immunofluorescence antibody staining for PCV2-infected PAMs.** Intracytoplasmic bright green fluorescence (fluorescein isothiocyanate) is seen in PCV2-infected PAMs. (**A**) Bright microscopy image of mock-inoculated PAMs. (**B**) Mock-inoculated PAMs. (**C**) PCV2-infected PAMs at 24 HPI. (**D**) PCV2-infected PAMs at 48 HPI. (**B**-**D**) were stained with PCV2 Cap protein-specific monoclonal antibody. Magnification: ×400.

Immunofluorescence assay (IFA) revealed green fluorescent cells in the infected PAMs, indicating that the PAMs were successfully infected by PCV2 at 24 and 48 hours after inoculation (Figure 1). There were no significant differences in the levels of viral DNA or virus particles between the two time points (mean viral loads: 5.67±0.35 log_10_ and 5.72±0.49 log_10_). Neither PCV2 particles nor the PCV2 nucleotide signal were detected in the mock-inoculated PAMs at any of the experimental time points.

### Transcriptome analysis of the PAM response to PCV2 infection

The concentrations of RNA ranged from 0.67 to 1.97 μg/μL. All RNA samples were considered to be of high quality, based on RNA integrity number (RIN) values (ranging from 8.0 to 9.4). The isolated mRNA samples from 24 HPI and 48 HPI PAMs hybridized to 6345 and 5856 probe sets on the transcriptome microarray, respectively corresponding to 34.25% and 31.65% of all probe sets (Additional file 1: Table S1). After quantile normalization and statistical analysis, 1277 transcripts were identified (false discovery rate (FDR)-adjusted *p*-value <0.05). Furthermore, the criteria of a two-fold or greater change in differential expression and a FDR of 5% were chosen to determine up-regulated and down-regulated genes. According to these criteria, 266 and 175 genes were differentially expressed (DE) from baseline at 24 HPI and 48 HPI (Additional file 2: Table S2 and Additional file 3: Table S3). According to the corresponding biological process Gene Ontology (GO) terms, or potential biological processes reported in recent publications, the majority of these genes were related to Immune Response, Inflammatory Response, Antigen Processing and Presentation of Peptide or Polysaccharide Antigen via MHC Class II, Anti-viral Immune response, Cell Motility, and Negative Regulation of Apoptosis (Table 1). However, the differential expression between 24 HPI and 48 HPI reached the threshold of statistical significance for only six of those genes (Additional file 4: Table S4 and Additional file 5: Table S5). A total of 17 differentially expressed genes were selected to validate the microarray results by quantitative reverse transcription PCR (qRT-PCR) using the primers in Table 2. The qRT-PCR results confirmed, in general, the patterns of up-regulated and down-regulated expression of the 17 genes that was indicated by the microarray analysis (Table 3).

**Table 1:** Functional grouping of genes differentially expressed (≥2-fold and P < 0.05, n = 3) between PCV2-infected and mock-infected control

**Functional classification**	**Gene name**	**Gene symbol**	**24 HPI FC**	**Q-value, % (24hpi)**	**48 HPI FC**	**Q-value, % (48hpi)**	**GenBank ID**
Immune response							
	rho GDP dissociation inhibitor beta	ARHGDIB	2.16	0.41	1.21	13.11	NM_001009600
	CMRF35-like molecule 1	CLM1	2.35	0.95	1.38	13.11	AY457047
	cathepsin C	CTSC	2.12	3.06	1.74	11.46	NM_001814
	cathepsin S	CTSS	0.5	1.09	0.77	19.25	M90696.1
	Fc fragment of IgG low affinity II b receptor	FCGR2B	3.47	0.54	1.85	8.49	NM_001033013
	neutrophil cytosolic factor 4	NCF4	2.62	0	2.12	6.28	NM_000631
Humoral immune response							
	chemokine ligand 16	CCL16	3.85	4.64	3.24	4.93	NM_004590;
	CD74 molecule	CD74	3.94	0	3.43	0.58	NM_213774
	complement factor D	CFD	0.36	0.41	0.59	2.88	NM_001928
	C-type lectin domain family 2, member B	CLEC2B	2.83	1.62	2.10	1.09	NM_005127
Inflammatory response							
	chemokine ligand 3-like 3	CCL3L3	7.58	0	2.46	1.64	NM_001001437
	chemokine Ligand 5	CCL5	1.53	14.04	2.25	2.44	AK312212
	chemokine ligand 5	CXCL5	32.28	0	12.96	0	EU176356
	chemokine ligand 7	CXCL7	2.75	0.95	3.57	1.97	NM_213862.1
	interleukin-6	IL6	2.26	4.65	1.36	13.43	NM_214399.1
	S100 calcium binding protein A1	S100A1	0.31	0.41	0.40	0.71	NM_006271
	S100 calcium binding protein A12	S100A12	10.98	0	5.13	1.09	FJ263393.1
	S100 calcium binding protein A8	S100A8	12.96	0	6.83	0	FJ263391.1
	S100 calcium binding protein A9	S100A9	20.00	0	9.71	0	FJ263392.1
	serum amyloid A1	SAA1	14.59	0.54	1.82	24.35	NM_199161
	secreted phosphoprotein 1	SPP1	2.66	2.39	1.67	24.35	AK296035
	tumor necrosis factor, alpha-induced protein 6	TNFAIP6	6.54	0	3.26	0.58	NM_001159607
Lipid biosynthesis							
	Lysophosphatidic acid acyltransferase, delta	AGPAT4	2.34	0	1.48	0.58	NM_020133
	lysophosphatidic acid acyltransferase, zeta	AGPAT6	1.56	0.54	2.03	0	NM_178819
	alkylglycerone phosphate synthase	AGPS	2.41	0.95	1.46	32.28	NM_003659
	farnesyl-diphosphate farnesyltransferase 1	FDFT1	0.77	14.04	0.45	1.82	AK297868
	isopentenyl-diphosphate delta isomerase 1	IDI1	0.84	26.53	0.46	2.88	NM_004508
	inositol-3-phosphate synthase 1	ISYNA1	2.12	0.54	1.65	1.44	NM_016368
	stearoyl-CoA desaturase	SCD	2.46	0.54	3.37	8.49	AB208982
	triosephosphate isomerase 1	TPI1	3.00	0	2.11	3.33	NM_000365
Locomotion							
	CD97 molecule	CD97	1.50	3.06	2.17	1.64	NM_001784
	coronin, actin binding protein, 1A	CORO1A	2.64	0.54	1.26	4.65	NM_007074
	chemokine ligand 10	CXCL10	7.69	4.93	2.58	1.07	NM_001565
	Kallmann syndrome 1 sequence	KAL1	0.43	1.44	0.46	4.65	NM_000216
	mitogen-activated protein kinase 1	MAP2K1	2.03	0	1.17	1.7	NM_002755
	platelet factor 4	PF4	4.15	0	3.80	1.64	NM_002619
	phosphatidic acid phosphatase type 2B	PPAP2B	8.45	0.95	3.04	11.46	NM_003713
	prostaglandin-endoperoxide synthase 2	PTGS2	5.75	0	5.41	0	NM_001784
Lyase activity							
	aldolase C, fructose-bisphosphate	ALDOC	3.08	0	2.18	3.33	AK294157
	carbonic anhydrase XIII	CA13	0.41	0.82	0.40	0.71	NM_198584
	enolase 1, (alpha)	ENO1	2.24	0	1.80	4.94	AK298600
	N-acetylneuraminate pyruvate lyase	NPL	1.71	1.62	2.28	0	AK297017
	serine dehydratase	SDS	7.58	0	9.14	0	NM_006843
Antigen processing and presentation							
	Major histocompatibility complex, class II, DO alpha	DOA	2.29	0	1.42	13.11	NM_002119.3
	Major histocompatibility complex, class II, DO beta	DOB	17.85	2.39	3.78	1.64	NM_002120.3
	Major histocompatibility complex, class II, DQ alpha 1	DQA1	2.07	1.62	1.37	9.21	M33906.1
	Major histocompatibility complex, class II, DQ beta 1	DQB1	2.47	0	2.14	11.46	M32577
	Major histocompatibility complex, class II, DR alpha	DRA	7.87	0	3.69	1.08	NM_001134339
	Major histocompatibility complex, class II, DR beta 1	DRB1	5.83	0.95	2.17	1.17	AY770514.1
Signal transducer activity							
	guanine nucleotide binding protein (G protein), gamma 2	GNG2	1.94	0.41	2.26	0.42	NM_0530
	regulator of G-protein signaling 1	RGS1	1.02	58.23	0.47	4.25	NM_002922
	transforming growth factor, alpha	TGFA	2.79	0	1.54	14.86	AK312899
	wingless-type MMTV integration site family, member 6	WNT6	0.49	1.09	0.64	21.46	NM_006522
Antiviral immune response							
	chemokine ligand 8	CCL8	8.02	0	3.17	0	NM_214214.1
	extracellular matrix protein 1	ECM1	2.01	0.54	2.89	0	CB477333
	IFN-gamma	IFNG	0.49	0.41	3.18	0	NM_213948.1
	interleukin 18	IL18	2.58	0.95	5.84	19.25	NM_001562
	ISG15 ubiquitin-like modifier	ISG15	1.56	10.92	2.51	0	EU584557.1
	interferon-stimulated gene 20 kDa protein	ISG20	1.49	61.10	2.20	4.94	NM_002201
	vasoactive intestinal peptide	VIP	0.69	32.11	4.14	0	NM_194435
Apoptosis							
	BCL2/adenovirus E1B 19kDa interacting protein 3	BNIP3	3.69	0	2.61	2.88	U15174.1
	CD14 molecule	CD14	3.60	0	3.87	0	NM_001097445
	immediate early response 3	IER3	5.46	0.41	2.21	1.64	NM_003897
	lectin, galactoside-binding, soluble, 1	LGALS1	0.42	0.82	0.66	10.51	NM_002305
	interleukin 1 alpha	IL1A	21.31	0	8.47	0	NM_214029.1
	interleukin 1, beta	IL1B	18.69	0	10.33	2.88	M15330.1
	serine/threonine kinase 17a	STK17A	1.53	1.62	2	1.64	NM_00476
	tumor necrosis factor receptor superfamily, member 10d, decoy with truncated death domain	TNFRSF10D	2.26	0	2.21	61.11	NM_003840
	tumor necrosis factor alpha	TNFα	5.13	0.41	1.96	6.28	EU682384.1
Cell proliferation							
	Betacellulin	BTC	2.26	0.95	1.78	1.97	NM_001729
	epithelial membrane protein 1	EMP1	0.49	0.82	0.62	2.88	NM_001423
	fatty acid binding protein 3, muscle and heart	FABP3	1.88	0.95	2.57	3.09	NM_004102
	fms-related tyrosine kinase 1	FLT1	1.91	1.78	2.49	0	NM_002019
	interleukin 2 receptor, gamma	IL2RG	1.39	6.28	2.06	1.07	NM_000206
	interleukin 8 receptor, beta	IL8RB	1.33	45.16	3.41	0	NM_001557
	v-myc myelocytomatosis viral oncogene homolog	MYC	0.75	14.04	0.29	0.71	NM_002467
	pro-platelet basic protein	PPBP	2.75	0.95	3.57	1.97	NM_002704

Note: The DE genes are presented with putative functions assigned by the GO term and manual annotation. Many genes with multiple functions were only listed in one category according to specific biology processes. FC: fold change; FC≥2: up-regulation (infection/control); FC≤0.5: down-regulation.

**Table 2:** Priers used for qRT-PCR validation and additional expression profiling

**Gene**	**Primer sequence, 5′-3′**		**Amplicon length, bp**	**GenBank accession number**
HPRT-1 ^a^	Forward:	TCATTATGCCGAGGATTTGGA	90	DQ136030
	Reverse:	CTCTTTCATCACATCTCGAGCAA		
GAPDH ^b^	Forward:	TGCCAACGTGTCGGTTGT	120	NG_007073.2
	Reverse:	TGTCATCATATTTGGCAGGTTT		
CD14	Forward:	AGAGTTCAAAGAGTAGGGAA	86	NM_001097445.2
	Reverse:	AACGCTATCTGTCCTCAC		
GPNMB	Forward:	CCCTCACGAGCACCCTTGT	91	NM_001098584
	Reverse:	AAGAAGCCAACGAAGACCAGGAT		
ICAM1	Forward:	GCAAGAAGATAGCCAACCA	106	NM_000201.2
	Reverse:	TGCCAGTTCCACCCGTTC		
IFNγ	Forward:	AAAGATAACCAGCCCATTC	93	NM_213948.1
	Reverse:	GTCATTCAGTTTCCCAGA		
IL6	Forward:	CCTTCAGTCCAGTCGCCTTCTCC	97	NM_214399.1
	Reverse:	GCATCACCTTTGGCATCTTCTTCC		
IL8	Forward:	CACTGTGAAAATTCAGAAATCATTGTTA	105	NM_213867.1
	Reverse:	CTTCACAAATACCTGCACAACCTTC		
ISG15	Forward:	GGAGGGTAGGGAGGAGGGA	108	EU584557.1
	Reverse:	ATGGGCGTCACACAGGCT		
S100A1	Forward:	GGGAGAACAGTTGAGCAGATGGT	104	NM_006271.1
	Reverse:	GGAGGGAACAGCGGGATGAA		
S100A12 ^c^	Forward:	GGCATTATGACACCCTTATC	168	NM_001160272.1
	Reverse:	GTCACCAGGACCACGAAT		
S100A4	Forward:	GGGGAAAAGGACGGATGAAGC	96	XM_001929560
	Reverse:	GCAGGACAGGAAGACGCAGTA		
S100A8 ^c^	Forward:	GCGTAGATGGCGTGGTAA	155	FJ263391.1
	Reverse:	GCCCTGCATGTGCTTTGT		
S100A9 ^c^	Forward:	CCAGGATGTGGTTTATGGCTTTC	186	NM_001177906.1
	Reverse:	CGGACCAAATGTCGCAGA		
TLR1 ^b^	Forward:	TGCTGGATGCTAACGGATGTC	102	NM_001031775.1
	Reverse:	AAGTGGTTTCAATGTTGTTCAAAGTC		
TLR2 ^b^	Forward:	TCACTTGTCTAACTTATCATCCTCTTG	162	AB085935.1
	Reverse:	TCAGCGAAGGTGTCATTATTGC		
TLR3 ^b^	Forward:	AGTAAATGAATCACCCTGCCTAGCA	112	DQ647698.1
	Reverse:	GCCGTTGACAAAACACATAAGGACT		
TLR4 ^b^	Forward:	GCCATCGCTGCTAACATCATC	108	AY535422.1
	Reverse:	CTCATACTCAAAGATACACCATCGG		
TLR5	Forward:	CTCGCCCACCACATTA	156	FJ668383
	Reverse:	TGAGGGTCCCAAAGAGT		
TLR6 ^b^	Forward:	AACCTACTGTCATAAGCCTTCATTC	95	NM_213760.1
	Reverse:	GTCTACCACAAATTCACTTTCTTCAG		
TLR8 ^b^	Forward:	AAGACCACCACCAACTTAGCC	105	NM_214187.1
	Reverse:	GACCCTCAGATTCTCATCCATCC		
TLR9 ^b^	Forward:	CACGACAGCCGAATAGCAC	122	AY859728.1
	Reverse:	GGGAACAGGGAGCAGAGC		
TNFα	Forward:	TGGTGGTGCCGACAGATGG	102	EU682384.1
	Reverse:	GGCTGATGGTGTGAGTGAGGAA		
VCAM1	Forward:	TCAGGGAGGACACAAAGAAGGG	135	NM_213891.1
	Reverse:	AAACGGCAAACACCATCCAAAGT		

^a^ from reference [21], ^b^ from reference [22], ^c^ from reference [9].

**Table 3:** qRT-PCR validation of representative genes that are differentially expressed between control and PCV2-infected PAMs

**Gene**	**GenBank ID**	**24 HPI**		**48 HPI**	
		**Microarray FC**	**qRT-PCR FC**	**Microarray FC**	**qRT-PCR FC**
CD14	NM_001097445.2	3.60	2.35(0.041)	3.87	2.71(0.003)
GPNMB	NM_001098584	−2.13	−4.04(<0.0001)	−2.58	−7.89(0.002)
ICAM1	NM_000201.2	4.02	3.59(<0.0001)	3.20	2.21(0.003)
IFNγ	NM_213948.1	−2.04	−2.76(0.005)	3.18	5.09(0.006)
IL6	NM_214399.1	2.26	4.60(0.001)	1.36	2.47(0.001)
IL8	NM_213867.1	4.86	8.65(0.001)	5.03	5.36(0.008)
ISG15	EU584557.1	1.56	1.37(0.006)	2.51	2.83(0.031)
S100A1	NM_006271	−3.20	−4.42 (0.001)	−2.49	−1.87 (0.01)
S100A12	NM_001160272.1	10.98	8.49(0.006)	5.13	3.98(0.005)
S100A4	XM_001929560	−3.14	−4.26(0.002)	−1.98	−3.27(0.001)
S100A8	FJ263391.1	12.96	15.47(<0.0001)	6.83	4.34(0.003)
S100A9	NM_001177906.1	20.00	16.56(0.007)	9.71	6.21(0.025)
TLR1	NM_001031775.1	2.76	2.29 (0.032)	5.83	4.89(0.016)
TLR8	NM_214187.1	−4.35	−3.01(0.002)	−1.14	−1.72(0.004)
TLR9	AY859728.1	2.47	4.66(0.012)	1.43	2.24(0.0001)
TNFα	EU682384.1	5.13	5.36(0.016)	1.96	2.53(0.022)
VCAM1	NM_213891	−2.33	−7.54(0.001)	−2.90	−4.83 (0.005)

All reactions were performed in triplicate. FC: fold-change.

### Functional GO analysis of DE genes in PCV2-infected PAMs

The Ingenuity pathway analysis (IPA) program was used to determine the relevance of PAM gene functionalities and gene networks associated with PCV2 infection. While 266 (24 HPI) and 175 (48 HPI) DE genes were imputed into IPA, only 173 and 124 were characterized with specific cellular functions.

The 173 DE genes from 24 HPI PAMs were placed into 19 functional groups (Additional file 6: Table S6 and Additional file 7: Figure S8). The most frequently represented category of gene functionality was Inflammatory Response (Cellular Movement, and Immune Cell Trafficking) with 22 genes, followed by Cell-To-Cell Signaling and Interaction, Cellular Growth and Proliferation, and Cellular Compromise.

The 124 DE genes from 48 HPI PAMs were placed into 16 functional groups (Additional file 8: Table S7 and Additional file 9: Figure S9). The most frequently represented category of gene functionality was also Inflammatory Response with 25 genes, followed by Antigen Presentation, Cellular Movement, Lipid Metabolism, Cell Cycle, and Cellular Growth and Proliferation.

### qRT-PCR validated DE genes in PCV2-infected PAMs

**Figure 2:**
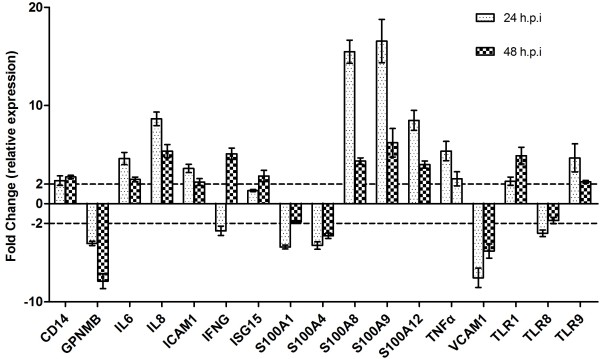
**Validation of microarray data by qRT-PCR analysis.** Fold-changes of gene expression in the PAMs following PCV2 infection compared with the control group are shown. The fold-changes were calculated by the formula of 2^-^ΔΔ Ct. Data is presented as mean ± SD of triplicate reactions for each gene transcript.

In order to validate the differential expression of some of the DE genes identified by microarray, eleven up-regulated genes and six down-regulated genes were selected for qRT-PCR analysis. All the selected genes were successfully amplified and the expression patterns corresponded to those observed by microarray. Although the extent of fold-change (FC) varied between the qRT-PCR and microarray results (Table 3 and Figure 2), the differential expression patterns were coincident, indicating the reliability of the microarray analysis.

### Toll-like receptors pathway analysis

Activation of the innate immune response is controlled in large part by the TLR family of pattern-recognition receptors (PRRs). A previous study showed that the DNA of PCV2 could impair the plasmacytoid- and monocyte-derived dendritic cells ability to induce interferon (IFN)-α and TNF-α by interacting with TLR7 and TLR9 [23]. In the current study, we verified the expression of TLR1, TLR2, TLR3, TLR4, CD14, TLR5, TLR6, TLR8, and TLR9 (the primers are listed in Table 2. Surprisingly, the two time points examined showed significant differences in up-regulation of TLR1 (2.29-fold vs. 4.89 fold), TLR4 cofactor CD14 (2.35-fold vs. 2.71-fold) and TLR9 (4.66-fold vs. 2.24-fold), and down-regulation of TLR8 (3.01-fold vs. 1.72-fold).

### Profile of PAM secreted cytokines in response to PCV2-infection

**Figure 3:**
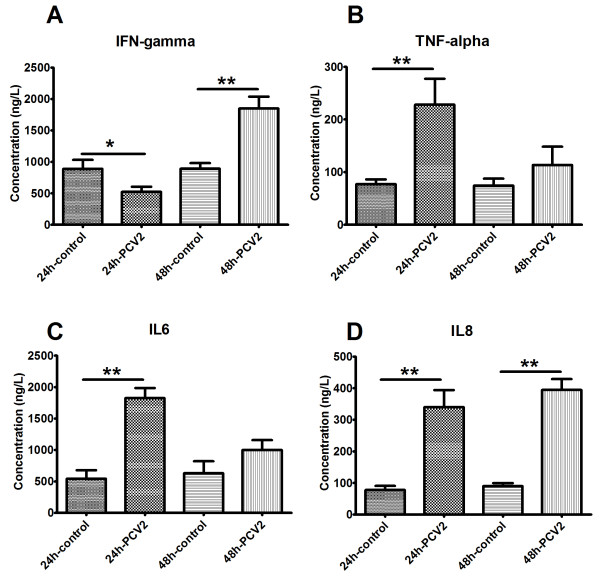
**Validation of microarray data by ELISA.** PCV2-induced changes in secreted levels of IFNγ (**A**), TNFα (**B**), IL-6 (**C**) and IL-8 (**D**). The protein levels in culture supernatants of mock-inoculated and PCV2-infected PAMs were measured by ELISA. Data are expressed as ng/L of three independent experiments. * 0.01 <p <0.05 and ** p<0.01 for PCV2-infected vs. mock-inoculated groups for the corresponding HPI.

When compared with the supernatant of mock-inoculated PAMs, PCV2-infected PAMs secreted significantly higher levels of interleukin (IL)-8 (340.09 ± 53.64 vs 77.43 ± 13.15, by 4~6-fold; Figure 3D). Tumor necrosis factor-alpha (TNFα) (228.13 ± 49.36 vs 77.12 ± 9.10) and IL-6 (1827.02 ± 157.32 vs 542.70 ± 137.05) levels were significantly higher in supernatants of PCV2-inoculated PAMs at 24 HPI (2~3-fold vs. mock-inoculated PAMs; Figure 3B-C). Interferon-gamma (IFNγ), however, appeared to be significantly down-regulated (552.87 ± 83.25 vs 889.99 ± 141.03, by 1.70-fold) at 24 HPI but significantly up-regulated (1848.10 ± 190.19 vs 892.37 ± 89.66, by 2-fold) at 48 HPI (vs. mock-inoculated; Figure 3A). The relative mRNA levels of porcine IL-8, TNFα, IL-6, and IFNγ showed the same DE trends as the secreted protein levels (Table 3).

## Discussion

PCV2 is an important pathogen of swine that has effects on both the pork industry and animal welfare. Despite the high mortality rates and widespread incidence of this pathogen, a definitive cure has not yet been found. An effective vaccine was created by Merial in 2005 (Circovac®; Merial, France) [24], and entered into widespread use in 2009. Despite the fact that the current vaccine strategy can efficiently control PCV2 infection, immunization failures exist in the field, and the molecular mechanisms underlying PMWS and porcine dermatitis and nephropathy syndrome (PDNS) caused by PCV2 remain largely unknown. The present study aimed to identify genes involved in the immune response against PCV2 in primary alveolar macrophages. Furthermore, by generating a comprehensive transcriptomic profile of the temporal PCV2 pathogenic process in the target host cells, we hoped to gain insights into the underlying molecular interactions and signaling pathways that may represent novel targets of improved preventative and therapeutic strategies.

### Toll-like receptors

Toll-like receptors are essential sensors of microbial infection that are involved in the recognition of a variety of microbial products [25-27]. To date, 13 TLRs have been identified and characterized for their particular cognate ligands and signaling cascades to trigger pathogen-targeted immune responses [28]. For example, TLR7 recognizes guanosine- and uridine-rich sequences derived from ssRNA viruses [29], while TLR9 recognizes single-stranded DNA (ssDNA) in endosomal compartments and signals to induce the production of type 1 interferons (IFNs) and pro-inflammatory cytokines and chemokines; PCV2 DNA has been shown to modulate cytokine secretion thorough interaction with TLR9 [30]. In our study of the circular, single-stranded DNA virus, PCV2, differential gene expression of TLRs was found. Specifically, PCV2 infection of PAMs led to increased expression of TLR1, TLR4 cofactor CD14, and TLR9. Monocytic CD14 signaling is usually activated upon exposure to bacterial LPS, and this molecule’s up-regulation may induce ineffective antibacterial immunity. In addition, a similar mechanism has been demonstrated in human monocytic cells upon exposure to the severe acute respiratory syndrome (SARS) coronavirus [31]. These receptors’ up-regulation may contribute to the increased sensitivity of macrophages to other pathogens, thereby augmenting the inflammation and host immune response. Such an enhanced immune response may be beneficial, but an over-stimulation of the innate response could result in tissue damage. For example, the same phenomenon was observed upon macrophage exposure to tobacco smoke, wherein the expression of TLR3 was increased and severe inflammation was observed after treated with TLR3 ligand [32].

### MHC class II molecules

MHC class II molecules play a central role in the initiation of the immune response by binding and presenting immunogenic peptides to CD4^+^ T_h_ lymphocytes. However, only a few cell types express MHC class II molecules, such as the antigen presenting cell types that include macrophages, B lymphocytes, Langerhans cells, and dendritic cells. The very complex and tight regulation of MHC class II gene expression has direct implications for T lymphocyte activation. These molecules include DOA, DOB, DQA1, DQB1, DRA, and DRB1, and their molecule chaperone CD74; all of which were up-regulated in PCV2-infected PAMs. This expression pattern contradicts the host response observed for other viruses, such as African swine fever virus (CSFV) [33], classical swine fever virus [34] and influenza A virus [35], and that was observed in follicles and PAMs of PMWS piglets [36,37]. Increased expression of MHC class II molecular have been shown to be correlated to increased interferon production in pigs infection infected with hog cholera virus [38]. IFN-γ up-regulation have been shown after 48 HPI, its up-regulation could benefit PCV2 replication [39]. The aberrant expression of class II antigens could also compromise production of immunoregulatory cytokines, compromising immune function and perhaps favor viral persistence through escape from immune recognition.

### Apoptosis and growth arrest

Apoptosis is considered to be an important host mechanism that interrupts viral replication and eliminates virus-infected cells. Viruses often kill infected cells by inducing apoptosis rather than necrosis, but repress apoptosis to prolong the life of the cell and increase the yield of progeny virions. SERPINB9, also called PI-9, belongs to the large superfamily of serine proteinase inhibitors, which bind to and inactivate serine proteinases, and acts to protect against granzyme B-mediated apoptosis [40]. PCV2-infected PAMs were found to have enhanced SERPINB9 expression, which may serve to prevent apoptosis and allow for longer-term replication and more virion production. BNIP3, an apoptosis-inducing dimeric mitochondrial protein [41], was also found to be increased in PCV2-infected PAMs. It is possible that this factor may represent the host’s counter response to PCV2, by which host-induced apoptosis would act to limit the amount of infected cells. It is also possible that these BNIP3 overexpressing cells represent a subset of PAMs that have reached full capacity of virus load, in which the virus itself may have manipulated the host gene expression to facilitate cell death and release of the virus progeny.

Actin alpha 2 (ACTA2) is a smooth muscle α-actin, whose expression is transformation-sensitive to growth signals in normal cells. Actively proliferating fibroblasts and smooth muscle cells have low levels of ACTA2, but the inhibition of cell proliferation by density arrest or treatment with antimitotic agents induces the smooth muscle α-actin promoter [42]. Thus, the ACTA2 up-regulation that was detected in PCV2-infected PAMs may be the result of growth arrest induced by the virus The same phenomenon was found in Rhabdomyosarcoma cells after EV71 infection [43].

ENO1 is involved in the induction of cell death in fibroblasts and has been implicated in tumorigenicity of human breast carcinoma cells [44]. Its regulation of cell growth is further suggested by the presence of functional transcriptional repression domains [45]. PCV2-infected PAMs showed enhanced ENO1 expression. It is possible that up-regulation of this gene may enhance growth arrest in PCV2-infected cells, thereby culminating in cell death.

### Inflammatory response

Serum amyloid A 1 (SAA1) is a member of the SAA family of apolipoproteins synthesized in response to cytokines released by activated macrophages, which often serve as clinical markers of acute and chronic inflammatory diseases [46]. The pattern of increased SAA1 expression observed in PCV2-infected PAMs suggested that PCV2 induced an acute inflammatory response at the early time point (24 HPI) of infection. TNF-α and IL-1β are acute-phase proinflammatory mediators that promote inflammation and induce fever, tissue destruction, and, in some cases, shock and death [47]. Our study showed that PCV2 could induce TNF-α and IL-1β expression (Table 1) in PAMs at 24 HPI and 48 HPI. These results are in good agreement with those from a previous study of lymphoid tissues and peripheral blood mononuclear cells (PBMCs) of PMWS pigs [17,48]. SAA1, TNF-α, and IL-1β up-regulation after PCV2 infection suggests the occurrence of systemic inflammation following PCV2 infection.

Lymphohistiocytic to granulomatous pneumonia is a common histopathological finding in naturally-acquired PMWS and experimentally-infected PCV2 pigs [49,50]. Chemokines, such as MCP-1/CCL-2, were found to be up-regulated in the PCV2-infected PAMs. In a previous study, MCP-1 was suggested to play an important role in the pathogenesis of granulomatous inflammation in PMWS-affected pigs [51-53]. The progressive granulomatous inflammation has been speculated to be compromise organ function and result in PMWS-associated fatalities [54]. Furthermore, the MCP-1 cytokine is known to be an important chemokine for the recruitment of monocytes from the blood [55]. Thus, macrophage infiltration has been suggested as an important feature of the pathogenesis and progression of PMWS [56].

Recruitment of inflammatory cells in injured tissue is regulated by various cytokines and chemokines. PMWS-affected pigs present with increased expression of MIP-1, CCL-2, IL-1β, IL-8, and TNF-α mRNA in the lymphoid tissues and PBMCs, suggesting that such cytokines and chemokines may contribute to the development of granulomatous lymphadenitis and recruitment of macrophages or other inflammatory cells to the PCV2-infected tissues [17,52,57]. The PCV2-infected PAMs in our study showed up-regulation of MIP-1, CCL-2, IL-1β, IL-8 and TNF-α mRNA expression. TNF-α is known to activate microvascular endothelium and cause a pyrexic response [47]. Increased production of TNF-α may induce fever and respiratory distress in PCV2-infected pigs [58,59]. IL-8 is a chemoattractant for neutrophils and other polymorphonuclear leukocytes (PMNs) that are produced following acute infection [60]. Interaction of IL-8 and MCP-1/CCL-2 is also known to trigger tight adhesion of monocytes to vascular endothelium under flow conditions [61]. The ability of PCV2 to induce IL-8 production in PAMs or PBMCs has been previously reported [57,62,63]. Similar to IL-8, the PAM-derived chemokine AMCF-II/CXCL5 [64] is able to recruit PMNs to a lesion area [65]. Unlike the PMN-derived chemoattractants discussed above, MCP-1 is a powerful chemoattractant for monocytes and its activities can be further enhanced by IL-8 [61]. It is reasonable to speculate that the development of interstitial pneumonia in pigs suffering from naturally-acquired PMWS or experimental infection of PCV2 may result from the recruitment of acute phase inflammatory cells and subsequent mononuclear phagocytic cells from blood vessels to interalveolar septa and alveolar spaces by up-regulated cytokines and chemokines released from PCV2-containing PAMs.

### Antiviral immune response

IFNγ is a Th1-specific cytokine produced by macrophages, NK cells, and other cell types during the onset of viral infection. It has a potent antiviral property that contributes to the control of acute viral infections, and is an important mediator of cellular responses. IFNγ mRNA was found to be slightly down-regulated at the early stage of *in vitro* PCV2 infection (24 HPI) of PAMs. This repressed expression may be indicative of suppressed Th1 responses, which may facilitate viral persistence and delayed viral clearance. At the late stage of *in vitro* infection (48 HPI), however, the IFNγ expression level increased. Such an up-regulation of expression may indicate a virus-modulated mechanism to enhance infection and replication at the late stage of PCV2 infection *in vivo*[39].

## Conclusions

This is the first study to evaluate the gene expression profile of PCV2-infected PAMs *in vitro*. Microarray analysis showed that expression of 266 and 175 PAM genes was altered after 24 and 48 hours of PCV2 infection, respectively. Among these, several genes related to the inflammatory response were up-regulated, suggesting that PCV2 may induce systemic inflammation. Collectively, this work established a comprehensive differential transcription profile of early and late PCV2 infection in PAMs, which revealed that PCV2 can induce persistent cytokine production mainly through the TLR1 and TLR9 pathways.

## Methods

### Virus and cells

The PCV2-WH strain (GenBank accession number: FJ870967) was isolated from pooled samples of spleen and lymph nodes of a PMWS-affected pig. The virus was amplified by passaging in PCV-free porcine kidney (PK-15) cells and confirmed as PCV2 by nucleotide sequence analysis and reactivity with PCV2 Cap protein-specific monoclonal antibody [66]. The titer of the PCV2 inoculum was 1.0×10^7^ TCID_50_/mL, as determined by titration in PCV-free PK-15 cells.

All animals’ tissue collection procedures were performed according to protocols approved by the Hubei Province PR China for Biological Studies Animal Care and Use Committee. Three 3-week-old Large White piglets were obtained from a herd in Hubei province, which tested negative for both PCV2 and porcine reproductive and respiratory syndrome virus (PRRSV), and were raised in controlled lab conditions. For the first three days of containment, the pigs were administered daily doses of enrofloxacin (1 mL of 5% solution) and lincospectin/spectinomycin (1 mL of 5 or 10% solution) to eliminate any residual bacterial pathogens [67]. Seven days later, the piglets were sacrificed. PAMs were collected by bronchoalveolar lavage and frozen in liquid nitrogen, as previously described [68]. Prior to use, the cells were confirmed as negative for PCV1, PCV2, parvovirus, pseudorabies virus (PRV), classical swine fever virus (CSFV), and PRRSV variously by PCR and RT-PCR [69,70]. The PAM phenotype was confirmed by flow cytometric detection of the macrophage markers SWC3, CD169, and SLAII [68].

### RNA isolation and IFA

PAMs were thawed and cultured for 48 h before treatment, as previously described [71,72]. One primary culture from each animal was split into two for experimental analysis. The first was infected with PCV2-WH from the 11th passage at a multiplicity of infection (MOI) of 1. The second was mock-inoculated with DMEM to serve as a control. Virus uptake by the cells was determined by IFA using a PCV2 Cap protein-specific monoclonal antibody [66] and by quantitative real-time PCR (qPCR) [73]. Briefly, DNA was extracted from the cells using the E.Z.N.A.™ Viral DNA Kit (OMEGA, USA) according to the manufacturer’s instructions. The amplification was performed in a 25 μL reaction mixture containing 12.5 μL of 2× THUNDERBIRD Probe qPCR Mix (TOYOBO, Japan), 9.45 μL sterile, nuclease-free water, 10 pmol of forward primer (5′-CCAGGAGGGCGTTCTGACT-3'), 10 pmol of reverse primer (5′-CGTTACCGCTGGAGAAGGAA-3′), 4 pmol of *Taq*Man probe (5′-AATGGCATCTTCAACACCCGCCTCT-3′), 0.05 μL of 50× ROX reference dye, and 2 μL of the extracted DNA. The reaction was run in a real-time thermocycler (7500 PCR System; Applied BioSystems Inc.) with the following conditions: 1 cycle at 50°C for 2 min, 1 cycle at 95°C for 10 min, and 45 cycles at 95°C for 15 s and 62°C for 60 s (real time).

At 24 HPI and 48 HPI, the cells were collected for RNA extraction with the TRIzol reagent (Invitrogen Life Technologies, USA). The extracted RNA was further purified with an RNA clean-up kit (Macherey-Nagel, Germany) and applied to a Bioanalyzer 2100 spectrophotometer (Agilent Technologies, USA) to assess the integrity, quality, and quantity of the sample. The corresponding supernatants were collected for subsequent protein analysis and stored at −80°C until use.

### RNA labeling, microarray hybridization, and data analysis

RNA was converted to aminoallyl-RNA with the Amino Allyl II MessageAmp™ aRNA Amplification Kit (Ambion, USA) and labeled with either Cy3 or Cy5 (Reactuve Dye Pack; Amersham, Sweden). Each Cy3- (or Cy5-) coupled PAM sample was then hybridized to its corresponding Cy5- (or Cy3-) coupled reference sample anchored on a pig microarray slide containing 20,400 oligonucleotides (US Pig Genome Coordination Program; http://www.animalgenome.org/pig/resources/array_request.html). Hybridizations and analyses were carried out according to a previously published protocol from CapitalBio Corporation (Beijing, China) [74]. Raw data was extracted from the TIFF images using LuxScan 3.0 software (CapitalBio). A spot-exclusion method was adopted to filter out faint spots, with signal intensities in the lowest 50%, and exclude the corresponding genes from further analysis. An intensity-dependent LOWESS program in the R language package was used to normalize the two-channel ratio values. As a measure of technical replication, each experiment was conducted with the corresponding dye swap.

Gene Cluster 3.0 and Eisen’s Treeview software (Stanford University, USA) were used to compare similarities among individual samples. Cy3/Cy5 ratios were log-transformed (base 2), median centered by arrays and genes, and hierarchically clustered (average linkage correlation metric). To determine gene products with significantly up-regulated expression, the PAM 24 HPI data sets were assessed for increases in average intensity of at least 2-fold. Statistical comparisons were performed by the one-class method in the significance analysis of microarray (SAM software version 3.0, Stanford University) [1]. All the microarray results from this study were deposited in the NCBI Gene Expression Omnibus database under the following accession numbers: platform, GPL13968; Series, GSE30918; Samples, GSM766444, GSM766445, GSM766446, GSM766447, GSM766448, GSM766449.

### Functional interpretation of microarray data as well as pathway and network analysis

Differentially expressed genes were selected for pathway exploration using the IPA web-based query system (Ingenuity Systems, http://www.ingenuity.com), this system can generate the functional analysis and networks that are most significant to the data set, IPA compares proteins in the input group and displays a rank-ordered list of pathways and networks, whose activities are most likely affected. Ficher’s exact test was used to calculate a p-value determining the probability that each biofunction assigned to that data set is due to chance alone. Genes from the dataset that met the fold change cut-off of 2.0 were considered for the analysis. The score is the probability that a collection of proteins equal to or greater than the number in a network could be achieved by chance alone. A score of 3 indicates that there is a 1/1000 chance that the focus proteins are in a network due to random chance, therefore, score of 3 or higher have a 99.9% confidence of not being generated by random chance alone.

### Quantitative RT-PCR

Equal amounts of RNA (1 μg) from PAMs was used to generate cDNA with oligo (dT) primers using the SuperScript II RNase H Reverse Transcriptase Kit (Invitrogen, USA). Quantitative RT-PCR reactions were set up in triplicate using reagents from the SYBR Green I PCR Kit (Toyobo) and 10 μM primers (designed by the Beacon Designer 7.0 program, http://www.premierbiosoft.com; Table 2). The amplification reactions were performed on a LightCycler 480 (Roche) with the following conditions: 30 s at 95°C, 40 cycles of 95°C for 10 s, 60°C for 5 s and 72°C for 10 s. Subsequently, melting curve analysis and quantitative analysis of the data were performed using the Roche LightCycler 480 software version 1.5.0. Each sample was run in triplicate. Samples were normalized at each respective time point, using mock-infected PAMs as calibrators and the hypoxantine phosphoribosyltransferase (HPRT-1) and glyceraldehyde-3-phosphatedehydrogenase (GAPDH) as the reference genes.. The average threshold cycles of the replicates (each gene was detected three times) were used to calculate the fold-changes between PCV2-infected or mock-inoculated PAMs by using the formula of 2-delta-delta Ct described by Livak and Schmittgen [75].

### Cytokine detection in culture supernatant

The secreted protein levels of IFNγ, IL-6, IL-8 and TNFα were determined by testing culture supernatants with the respective commercial ELISA kits (R&D Systems, USA). All samples were tested in triplicate and read at 450 nm using an ELISA plate reader (Bio-Tek, USA).

### Statistical analysis of quantitative PCR data

Statistical analyses were carried out using Microsoft Excel 2007 (Microsoft Co., USA). Differences between groups were assessed by one-way repeated measures analysis of variance followed by Tukey’s multiple comparison tests. *P-values* less than 0.05 were considered to indicate statistical significance.

## Abbreviations

CSFV: Classical swine fever virus; DE: Differentially expressed; FC: Fold-change; FDR: False discovery rate; GO: Gene ontology; HPI: Hours post infection; IFNγ: Interferon-gamma; IL: Interleukin; IPA: Ingenuity pathway analysis; ORF: Open reading frame; PAMs: Porcine alveolar macrophages; PBMC: Peripheral blood mononuclear cell; PCR: Polymerase chain reaction; PCV: Porcine circovirus; PMN: Polymorphonuclear leukocytes; PRRSV: Porcine reproductive and respiratory syndrome virus; qPCR: Quantitative PCR; qRT-PCR: Quantitative reverse transcription PCR; TLR: Toll-like receptor; TNFα: Tumor necrosis factor-alpha.

## Competing interests

The authors declare that they have no competing interests.

## Authors’ contributions

LWT carried out most of the experiments and wrote the manuscript. HQG, CHC, CC, and JCA critically revised the manuscript and the experimental design. LSQ, WY, DF, YWD, and YK helped with the experiments. All of the authors read and approved the final version of the manuscript.

## Supplementary Material

Additional file 1: Table S1 PAM transcriptome analysis following PCV2 infection at 24 and 48 hours post-infection in comparation to the mock-infected control. Click here for file

Additional file 2: Table S2 DE transcripts (n=267) after PCV2 infection at 24 hours post-infection (q-value<5%, FC≥2.0 or ≤0.5). Fold changes represent the difference in expression levels in infected and mock-infected control. Click here for file

Additional file 3: Table S3 DE transcripts (n=175) after PCV2 infection at 48 hours post-infection, (q-value<5%, FC≥2.0 or ≤0.5). Fold changes represent the difference in expression levels in infected and mock-infected control. Click here for file

Additional file 4: Table S4 Six DE transcripts at 48 HPI, as compared to 24 hours post-infection (q-value<5%, FC≥2.0 or ≤0.5). Click here for file

Additional file 5: Table S5 Complete list of the DE transcripts at 24 and 48 hours post-infection (q-value<5%, FC≥2.0 or ≤0.5). Click here for file

Additional file 6: Table S6 Gene list of DE genes grouped by IPA based on gene function after 24 hours post-infection. The table contains columns with names of genes involved in the network, the score value and the top functions. Focus genes are shown in red. Click here for file

Additional file 7: Figure S8 Top networks of interacting genes from the DE genes at 24 hours post-infection analyzed by IPA. Click here for file

Additional file 8: Table S7 Gene list of DE genes grouped by IPA based on gene function after 48 hours post-infection. The table contains columns with names of genes involved in the network, the score value and the top functions. Focus genes are shown in red. Click here for file

Additional file 9: Figure S9 Top networks of interacting genes from the DE genes at 48 hours post-infection analyzed by IPA. Click here for file
