# Supplementary material for: Transcription analysis of the porcine alveolar macrophage response to porcine circovirus type 2
Source: BMC Genomics. 2013 May 27;14:353. doi: 10.1186/1471-2164-14-353 (PMC3680065; doi:10.1186/1471-2164-14-353)

**Supplemental Figure 8. Top networks of interacting genes from the DE genes at 24 HPI analyzed by IPA.**

A: Inflammatory Response, Cellular Movement, Immune Cell Trafficking;

B: Cell-To-Cell Signaling and Interaction, Hematological System Development and Function;

C: Free Radical Scavenging, Cell-To-Cell Signaling and Interaction, Cellular Compromise.


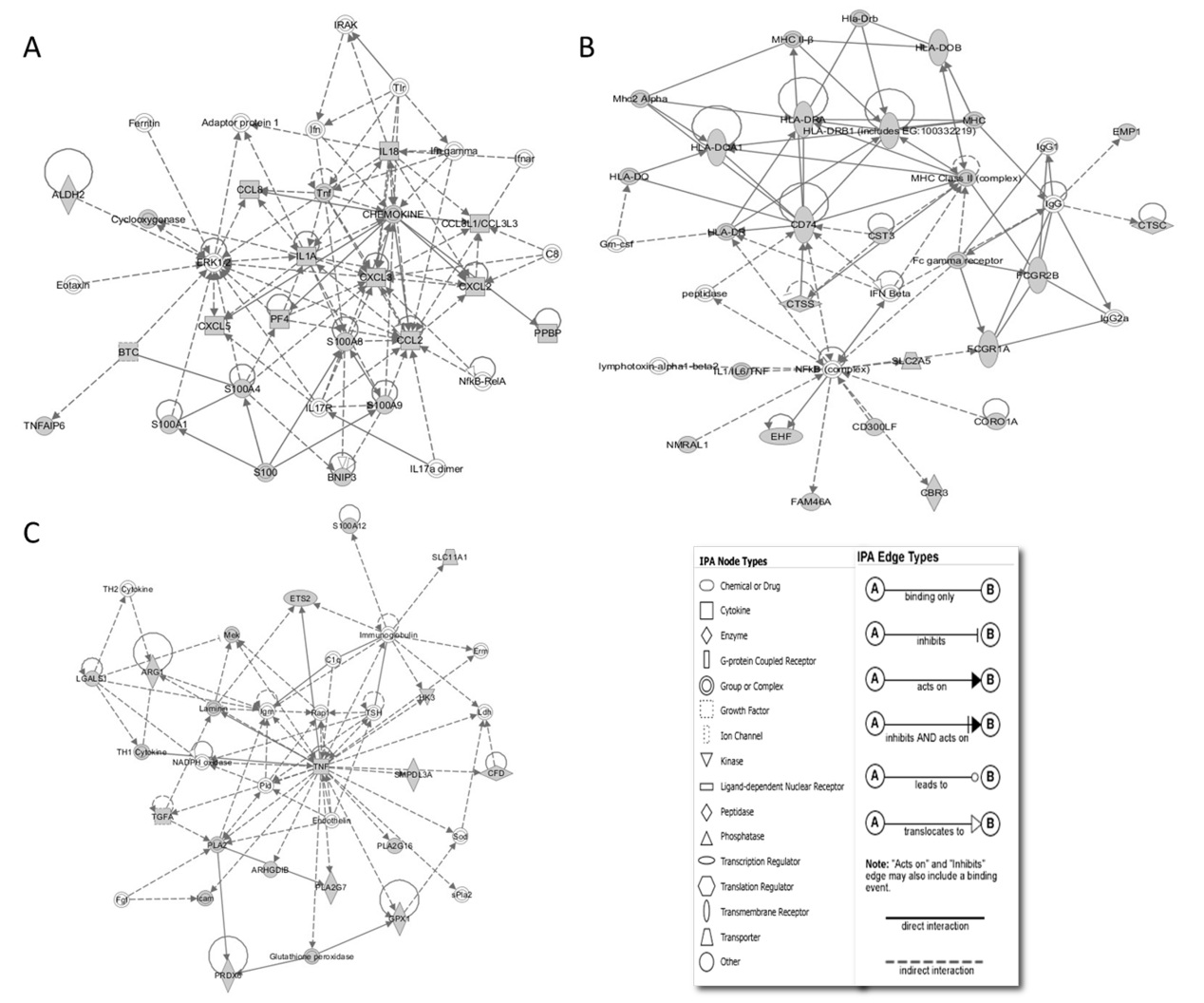

Supplement: Additional file 7: Figure S8 — Top networks of interacting genes from the DE genes at 24 hours post-infection analyzed by IPA. [file 1471-2164-14-353-S7.doc]
