# Supplementary material for: Transcription analysis of the porcine alveolar macrophage response to porcine circovirus type 2
Source: BMC Genomics. 2013 May 27;14:353. doi: 10.1186/1471-2164-14-353 (PMC3680065; doi:10.1186/1471-2164-14-353)

**Supplemental Figure 9. Top networks of interacting genes from the DE genes at 48 HPI analyzed by IPA.**

A: Inflammatory Response, Antigen Presentation, Cellular Movement;

B: Cell Cycle, Cellular Growth and Proliferation, Lipid Metabolism;

C: Drug Metabolism, Glutathione Depletion In Liver, Lipid Metabolism.


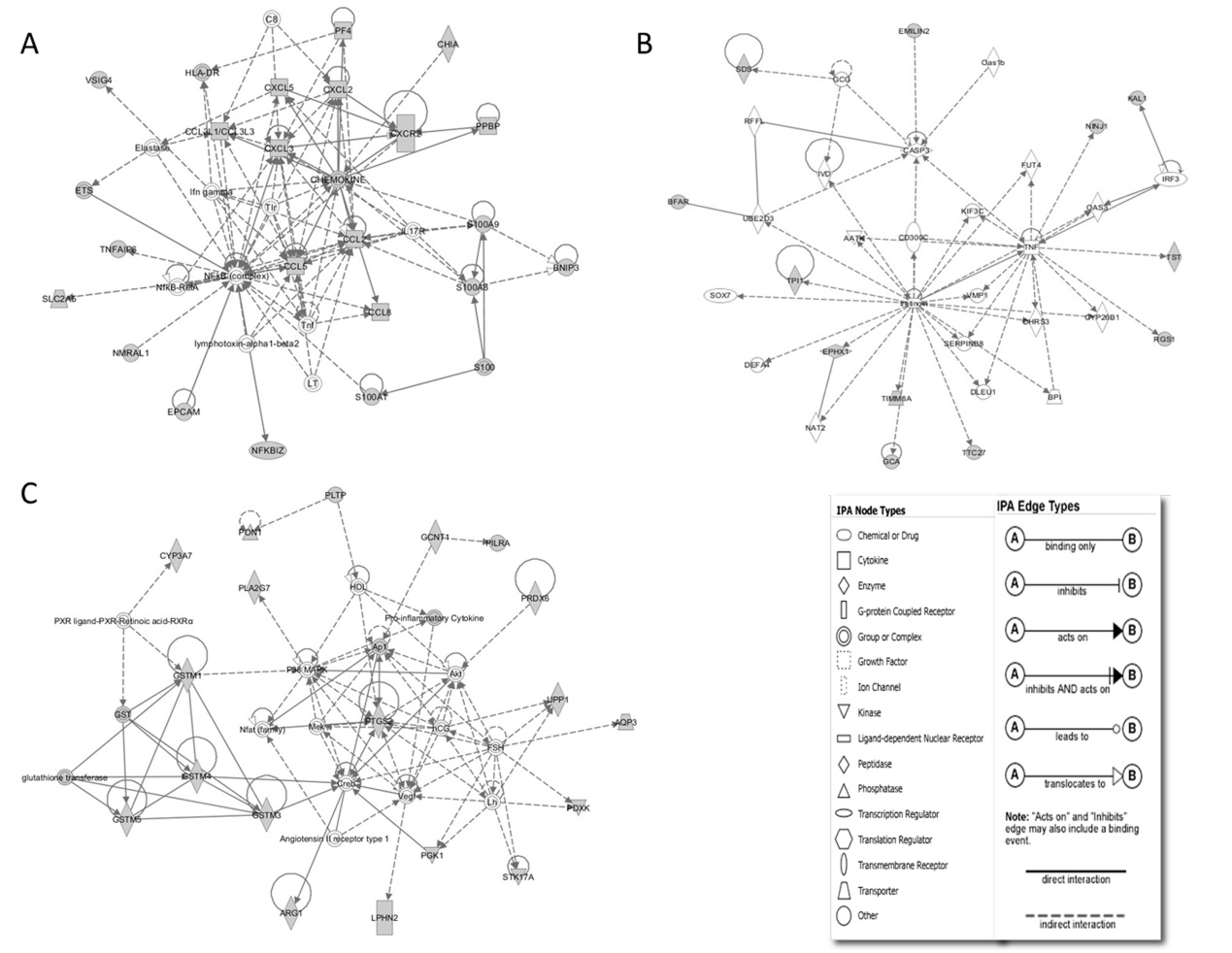

Supplement: Additional file 9: Figure S9 — Top networks of interacting genes from the DE genes at 48 hours post-infection analyzed by IPA. [file 1471-2164-14-353-S9.doc]
